# Supplementary material for: The obligate intracellular bacterium Orientia tsutsugamushi differentiates into a developmentally distinct extracellular state
Source: Nat Commun. 2022 Jun 23;13:3603. doi: 10.1038/s41467-022-31176-9 (PMC9226355; doi:10.1038/s41467-022-31176-9)
Supplement: Supplementary file 3 — Reporting Summary [file 41467_2022_31176_MOESM3_ESM.pdf]

nature portfolio

Corresponding author(s): Jeanne Salje

Last updated by author(s): Oct 5, 2021

Reporting Summary

Nature Portfolio wishes to improve the reproducibility of the work that we publish. This form provides structure for consistency and transparency in reporting. For further information on Nature Portfolio policies, see our Editorial Policies and the Editorial Policy Checklist.

Statistics

For all statistical analyses, confirm that the following items are present in the figure legend, table legend, main text, or Methods section.

n/a	Confirmed	
☐	☒	The exact sample size (n) for each experimental group/condition, given as a discrete number and unit of measurement
☐	☒	A statement on whether measurements were taken from distinct samples or whether the same sample was measured repeatedly
☐	☒	The statistical test(s) used AND whether they are one- or two-sided<br>*Only common tests should be described solely by name; describe more complex techniques in the Methods section.*
☐	☒	A description of all covariates tested
☐	☒	A description of any assumptions or corrections, such as tests of normality and adjustment for multiple comparisons
☐	☒	A full description of the statistical parameters including central tendency (e.g. means) or other basic estimates (e.g. regression coefficient) AND variation (e.g. standard deviation) or associated estimates of uncertainty (e.g. confidence intervals)
☐	☒	For null hypothesis testing, the test statistic (e.g. F, t, r) with confidence intervals, effect sizes, degrees of freedom and P value noted<br>*Give P values as exact values whenever suitable.*
☒	☐	For Bayesian analysis, information on the choice of priors and Markov chain Monte Carlo settings
☒	☐	For hierarchical and complex designs, identification of the appropriate level for tests and full reporting of outcomes
☒	☐	Estimates of effect sizes (e.g. Cohen's d, Pearson's r), indicating how they were calculated

Our web collection on statistics for biologists contains articles on many of the points above.

Software and code

Policy information about availability of computer code

Data collection	Licor Odyssey CLX imaging software v 3.1<br>Leica TCS SP8 confocal microscope software<br>Biorad CFX Maestro software v 3.1<br>Orbitrap Fusion Lumos mass spectrometer software<br><br>*since it is not easy to edit this version<br>all changes are made in the "clean" version
Data analysis	GraphPad Prism version 9.2.0<br>FIJI Image J software version 1.0<br>Proteome Discoverer version 2.3 Thermo Scientific

For manuscripts utilizing custom algorithms or software that are central to the research but not yet described in published literature, software must be made available to editors and reviewers. We strongly encourage code deposition in a community repository (e.g. GitHub). See the Nature Portfolio guidelines for submitting code & software for further information.

Data

Policy information about availability of data

All manuscripts must include a data availability statement. This statement should provide the following information, where applicable:
- Accession codes, unique identifiers, or web links for publicly available datasets
- A description of any restrictions on data availability
- For clinical datasets or third party data, please ensure that the statement adheres to our policy

Source data are provided with this paper as raw data files. Proteomics data is available on ProteomeXchange (PXD028218) and jPOST (JSPT001299)

Field-specific reporting

Please select the one below that is the best fit for your research. If you are not sure, read the appropriate sections before making your selection.

☒ Life sciences ☐ Behavioural & social sciences ☐ Ecological, evolutionary & environmental sciences

For a reference copy of the document with all sections, see nature.com/documents/nr-reporting-summary-flat.pdf

Life sciences study design

All studies must disclose on these points even when the disclosure is negative.

Sample size	No power analysis was undertaken to determine sample size. We used different samples sizes for different experiments and these are detailed in the figure legends accompanying each experiment. Samples sizes were chosen based on guidelines to meet requirements for statistical analyses.
Data exclusions	No data was excluded from the analysis
Replication	The number of independent replicates was always ≥3 and this is described for each experiment in the corresponding figure legend
Randomiza	When designing our experiments we ensured that similar numbers of cells were assigned to each group and treatment condition. Cells were imaged from multiple randomly chosen fields within each experiment. All cells within a field were selected for statistical analysis.
Blinding	Blinding was not carried out in this study due to the small number of conditions being compared which made it difficult for the experimentalist to lack knowledge of the conditions. In order to circumvent this, microscopy images within one experiment were acquired using constant settings and at least ten fields were randomly imaged per condition. Field randomization was achieved by locating regions of interest based on the Hoescht-stained nucleus, which was broadly unaffected by experimental perturbations, rather than selecting regions of interest based on bacterial labelling.

Reporting for specific materials, systems and methods

We require information from authors about some types of materials, experimental systems and methods used in many studies. Here, indicate whether each material, system or method listed is relevant to your study. If you are not sure if a list item applies to your research, read the appropriate section before selecting a response.

Materials & experimental systems

n/a	Involved in the study	
☐	☒	Antibodies
☐	☒	Eukaryotic cell lines
☒	☐	Palaeontology and archaeology
☒	☐	Animals and other organisms
☒	☐	Human research participants
☒	☐	Clinical data
☒	☐	Dual use research of concern

Methods

n/a	Involved in the study	
☒	☐	ChIP-seq
☒	☐	Flow cytometry
☒	☐	MRI-based neuroimaging

Antibodies

Antibodies used	rat anti-TSA56 monoclonal<br>rabbit anti-ScaC polyclonal<br>rabbit anti-ScaA polyclonal
Validation	All these antibodies were custom made for our group. They were validated using Western blot analysis and this data is available within the manuscript.

Eukaryotic cell lines

Policy information about cell lines

Cell line source(s)	L929 cells (ATCC CCL-1)<br>Primary Human Umbilical Vein Endothelial Cells (HUVEC) (ATCC PCS-100-010)<br>Both purchased from ATCC
Authentication	The cell lines have been authenticated by STR profiling by the vendor. No further authentication was performed.

Mycoplasma contamination	All cell lines tested negative for mycoplasma contamination
Commonly misidentified lines (See ICLAC register)	*Name any commonly misidentified cell lines used in the study and provide a rationale for their use.*
